# A Chaotic-Based Encryption/Decryption Framework for Secure Multimedia Communications

**DOI:** 10.3390/e22111253

**Published:** 2020-11-04

**Authors:** Ibrahim Yasser, Mohamed A. Mohamed, Ahmed S. Samra, Fahmi Khalifa

**Affiliations:** 1 Communications and Electronics Engineering Department, Nile Higher Institute for Engineering and Technology, Mansoura 35524, Egypt; ibrahimyasser14@gmail.com; 2 Electronics and Communications Engineering Department, Faculty of Engineering, Mansoura University, Mansoura 35516, Egypt; mazim12@mans.edu.eg (M.A.M.); shmed@mans.edu.eg (A.S.S.)

**Keywords:** chaotic maps, multimedia cryptosystems, audio data, image encryption, chaos encryption, security analysis

## Abstract

Chaos-based encryption has shown an increasingly important and dominant role in modern multimedia cryptography compared with traditional algorithms. This work proposes novel chaotic-based multimedia encryption schemes utilizing 2D alteration models for high secure data transmission. A novel perturbation-based data encryption for both confusion and diffusion rounds is proposed. Our chaotification structure is hybrid, in which multiple maps are combined combines for media encryption. Blended chaotic maps are used to generate the control parameters for the permutation (shuffling) and diffusion (substitution) structures. The proposed schemes not only maintain great encryption quality reproduced by chaotic, but also possess other advantages, including key sensitivity and low residual clarity. Extensive security and differential analyses documented that the proposed schemes are efficient for secure multimedia transmission as well as the encrypted media possesses resistance to attacks. Additionally, statistical evaluations using well-known metrics for specific media types, show that proposed encryption schemes can acquire low residual intelligibility with excessive nice recovered statistics. Finally, the advantages of the proposed schemes have been highlighted by comparing it against different state-of-the-art algorithms from literature. The comparative performance results documented that our schemes are extra efficacious than their data-specific counterpart methods.

## 1. Introduction

Rapid and increased growing of multimedia data exchange over open networks and the Internet necessitates reliable and robust security means to provide confidentiality and to prevent unauthorized access to the transferred content. Among several solutions is the employment of data encryption [1]. Encryption sachems are algorithms that modify data (such as text, image, sound, etc.) so that they are unreadable, invisible or impenetrable during transmission. Nowadays, data encryption plays an immense role in various applications and various encryption schemes have been developed with the ultimate goal to protect sensitive data by increasing its security and confidentiality [2].

Most of the research work aims at providing improved encryption quality, less execution, and security robustness against attacks. Compared with traditional encryption schemes, chaos-based schemes have demonstrated outstanding performance with proven ability of increased security and privacy needed by utilizing variable keys [3,4,5,6,7]. The basis is to use many components of chaos to decorate confusion and diffusion abilities of a given scheme [8]. In literature, chaos-based encryptions span a wide spectrum of multimedia types, i.e., text, audio, image, and video. Next, an overview of the recent literature work on a chaotic maps-based multimedia encryption application is provided.

Chaotic maps have been utilized by several groups for textual content encryption. In particular, Ekhlas et al. [9] proposed a textual content-encryption approach based on block cipher and chaotic maps. Their algorithm encrypted/decrypted an 8 × 8 bytes block primarily based on permutation and substitution the byte in S-box. Although their method employs large key space, it demonstrated low entropy and low security. A symmetric text cipher set of rules based totally on chaos was proposed by Murillo et al. [10]. Their scheme combined a mystery key of 128-bit length, optimized logistic maps with pseudo-random sequences, plain text characteristics, and optimal permutation diffusion spherical. The method demonstrated fast encryption speed; however, it has a small parameter space. Volos et al. [11] devised a textual content encryption process that is realized with a chaotic pseudorandom bit generator. The latter is based on two logistic maps with specific preliminary conditions and system parameters, running facet-by way of-side. The main advantage of the method in [11] is its simple realization using the X-OR function in the bit sequences.

Speech cryptography literature has recently shown an increased utilization of chaos theory as well. For example, a speech cryptographic method -based on a combination of permutation and substitution of speech samples using a chaotic Zaslavsky map was developed by Yousif et al. [12]. Their scheme demonstrated high-security features with low correlation. Jawad et al. [13] presented a two-level chaotic verbal encryption exchange system. Namely, a chaotic scrambling and protecting. Their algorithm provided stronger encryption with a very long key space; however, the scheme complexity was very high. Mahdi et al. [14] presented a voice chaotic-based protection model. Their technique utilized digital scrambling, using a duffing map, in which each speech sample is divided into eight bits. Similar to [13], the algorithm has a longer key space, but it showed reduced security performances.

On the other hand, chaotic-based image encryption has been investigated thoroughly in literature. For example, Wang et al. [15] offered a picture encryption technique utilizing hash function and cyclic shift. First, mask and diffusion operations were employed to exchange the pixel’s values. The final vicinity and value of pixels are altered by a bit cycle shift. Resistances to common attacks was the characteristic advantage of the method proposed in [15]. Wu et al. [16] introduced a photo encryption technique based totally on two-dimensional (2D) chaotic maps generated through the combining Henon and Sine maps. Amina et al. [17] proposed a new chaotic cipher approach specialized for medical images. Their technique included two stages: pixel diffusion and chaotic confusion. Both the methods in  [16,17] reported less encryption/decryption time; however, they also exhibit reduced compression capabilities. Another scheme was developed by Lou et al. [18] to shield picture contents while transferred over the internet. Their approach utilized Deoxyribonucleic Acid (DNA) approach and a 2D Henon-Sine map (2D HSM) for pixels’ diffusion and permutation. Although their method demonstrated low entropy analysis, it was highly compressible. Alwadi et al. [19] proposed a fast and hybrid system that cascades and combines chaotic maps. However, their method showed low robustness against some attacks. Yousif et al. [6] developed an encryption framework that utilizes a bank of chaotic maps, from which a correlation-based criterion is employed to select the candidate map for encryption. Their correlation analysis used all image pixels compared with literature schemes, which randomly chose a predefined number of neighboring pixel pairs (≈1000). Due to the nature of the initial map selection, their approach is more efficient for image encryption.

Video encryption has been also explored by various researchers. Particularly, Ganeshkumar et al. [20] provided a three-level chaos-video cryptosystem. They employed permutation and diffusion rounds, where the preliminary encryption parameters are obtained using a combination of logistic and tent (LTS) maps. Their method showed good time competency; however, compression reduction was its drawback. Valli et al. [21] presented video encryption pipeline that utilized S-Box and has two alternative schemes. The first is a higher-dimensional (i.e., 12D) chaos structure, and the second uses the Ikeda Delay Differential Equation (DDE). The latter was designed for real-time video encryption. The main limitations of their method are the increased encryption time and key complexity.

The overview of the related work revealed that chaotic systems have been exploited for efficient for multimedia data encryption. Traditional chaos-based cryptographic systems may be effective for text data; however, they fail in providing the same security level for the voice data. This is mainly due to the high redundancy and bulk data capacity of voice signals. Also, some chaos-based image encryption methods have security liabilities, such as resistance to chosen–plaintext attack, sensitivity to chaotic secret keys, the first pixel in the cipher image cannot be correctly restored, and there are additional restrictions on parameters selection for the inverse rectangular transform system.

To partially overcome the above-stated limitations and security defects, the work presented in this manuscript proposes improved multimedia encryption algorithms utilizing 2D alteration models. The main contributions of this work are: (i) novel multiple chaotic maps that depict high chaotic behavior for all parameter settings (i.e., different control parameter selections will lead to optimal chaotic behavior as no single chaotic region outperforms the others); (ii) perturbation-based data encryption are employed in both the confusion and diffusion rounds to outdo deficiencies of traditional architectures; (iii) the proposed chaotic techniques have high sensitivity to any change in its initial conditions, in addition to the other properties such as random behavior, ergodicity, and the long periodicity; (iv) the proposed chaotic systems combine both faster and secure encryption/decryption compared to conventional literature systems; (v) our pipeline is a comprehensive multimedia (textual content, voice, image, and video) security system.

The remainder of this paper is prepared as follows. In Section 2, the proposed scheme of multimedia security machine is clearly defined and the proposed chaotic maps of the cryptosystems are detailed. In Section 3, quantitative performance measurements that are used for evaluation are provided. Experiments and the associated results of the proposed cryptosystem, the comparisons with other well-known approaches, and security analysis results are detailed and discussed in Section 4. Eventually, the concluding remarks and venues for future research are given is Section 5.

## 2. The Proposed Multimedia Security System

**Figure 1:**
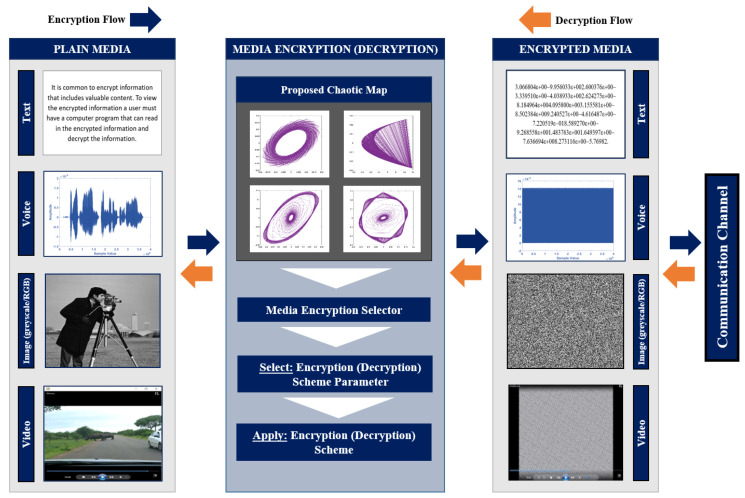
Schematic structure of the encryption and decryption processes of the proposed pipeline.

Unlike overall encryption techniques, careful strategies encrypt the most effective part of records to make the complete multimedia content material impenetrable. Our cryptosystem is depicted in Figure 1. The data portion that needs encryption is typically the essential/important records from either the very last bitstream or the intermediate steps. Encrypting this small quantity of critical records consumes less computational assets in comparison to the encryption of a huge quantity of unnecessary statistics, for attaining an identical stage of deterioration. Even though the identical principle is carried out in all approaches, they still differ on the basis of the encrypted information, followed standards to choose the important data, encryption approaches, and the domain and bitstream used. Here, several multimedia strategies are introduced for various data types (audio, image, etc.) and are thoroughly explained next.

### 2.1. The Proposed Cryptosystems

We developed a novel chaotic pipeline for enhancing encryption satisfaction and execution. Our machine is a 2D dynamical, nonlinear, discrete-time approach. For optimization algorithms in stochastic searching, the methodologies using chaotic factors rather than arbitrary factors are called chaotic optimization algorithm. Because of the non-repeatability and chaos ergodicity in these schemes, it can achieve overall searches at higher speeds than stochastic counterparts [22]. The chaotic sequence are derived from the Chirikov standard map model. Traditional chaotic maps are afflicted by low control parameters that cause a constrained chaotic range. On contrary, higher dimensional maps (like the proposed ones) may be used to increase the key space, immoderate complexity, and enhances the randomness of pseudo series. The characteristics of the four proposed maps are analyzed below.

In brief, the introduced maps have appealing characteristics, such as high sensitivity to initial conditions changes, ergodicity, random behavior, and the long periodicity. Those traits are comparable to the necessities of good encryption algorithms. Further, the proposed maps preserve the authentic structure of the classical maps in their parameter variety. In this work, we utilized four novel distinct maps. Namely, the finance, the modified logistic, the eye, and the galaxy maps. In those maps, an original position $\left(x_{n},y_{n}\right)$ is mapped to a new position $\left(x_{n+1},y_{n+1}\right)$ using a set of dynamical nonlinear equations, that are mathematically defined as follows:

(1) $$\begin{array}{ccc} y_{n+1} & = & y_{n}-\alpha \tan x_{n}\\ x_{n+1} & = & \sin x_{n}+y_{n+1} \end{array}$$

(2) $$\begin{array}{ccc} y_{n+1} & = & b^{2}x_{n}\\ x_{n+1} & = & {x_{n}}^{2}+{y_{n}}^{2}-a^{2} \end{array}$$

(3) $$\begin{array}{ccc} y_{n+1} & = & \tan y_{n}-\alpha \sin x_{n}\\ x_{n+1} & = & \sin x_{n}+\tan y_{n+1} \end{array}$$

(4) $$\begin{array}{ccc} y_{n+1} & = & \sin y_{n}-\alpha \tan x_{n}\\ x_{n+1} & = & \tan x_{n}+\sin y_{n+1}, \end{array}$$

where *x* and *y* are the simulated time series, α represents the external control parameter, and *n* is the iteration number. Equation (1) represents a new 2D discrete-time, dynamical, nonlinear finance model that reveals chaotic behavior. Moreover, Equation (2) is considered as 2D evolution of the logistic map that is similar to Henon map. This new map cogitates chaotic nature that is controlled with the external parameters *a* and *b*. As the name implies, Equation (3) represents a chaotic model that has the same shape of an eye. Finally, the fourth map is generated using the iterative function in Equation (4). It is worth mentioning that $x_{n}$ and $y_{n}$ are both deterministic time series and are ∈[0,1]. To create such maps, the steps outlined in Algorithm 1 should be followed.

**Figure 2:**
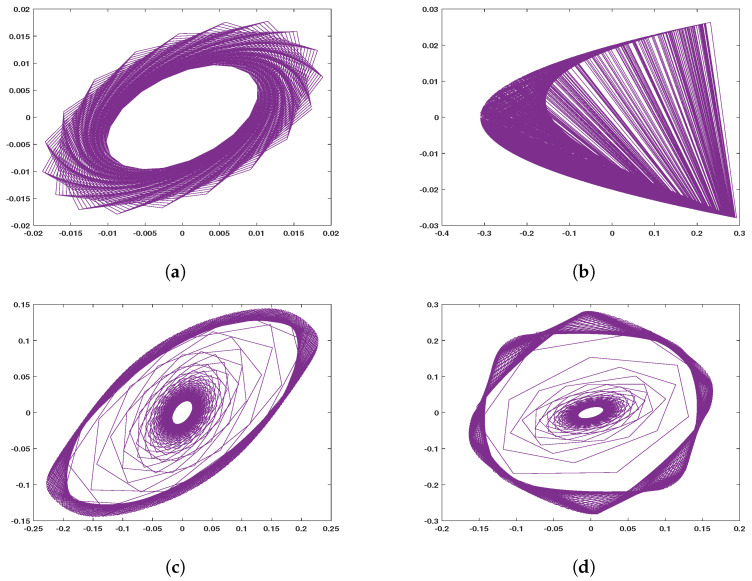
Two-dimensional (x,y) phase plot showing the dynamics of the proposed maps: (**a**) using Equation (1); (**b**) using Equation (2), a=1.4, and b=0.3, (**c**) using Equation (3), and (**d**) using Equation (4). Note that for all simulated maps $x_{\left(0\right)}=0.1$, $y_{\left(0\right)}=0.1$, α=0.9, and the number of iteration (n) is 1000.

Generally, distribution uniformity of chaotic behavior and the span of the parametric space of given chaotic maps can be examined using specific diagrams (i.e., bifurcation diagrams) to assure their suitability for the field of multimedia encryption [8]. Moreover, chaotic maps dynamics are specified by orbits, which are characterized via a non-smooth and discontinuous movement, see Figure 2. As may be effectively seen from the plots, each map exhibits a unique signature. The balance focuses on the introduced finance model are gotten via fathoming the above structures of equations. The characteristic types of the modified finance models can be achieved by Algorithm 1 using the control parameters, α=0.9, and $x_{\left(0\right)}=y_{\left(0\right)}=0.1$. The above maps are used for various multimedia encryption (image, video, audio, and text), which are discussed in the following subsections.
**Algorithm 1** Chaotic maps generation pseudocode.1. **Select** one of the models defined by Equations (1) through (4).2. **Define** model parameters: maximum number of iteration, upper and lower boundaries,  population size, number of dimensions and the fitness function.3. **Initialize** map positions randomly (i.e., $x_{\left(0\right)}$ and $y_{\left(0\right)}$).4. **Iterate**
*n* times: **update**
$x_{n+1}$ and $y_{n+1}$5. **End** iteration.

### 2.2. The Proposed Text Encryption/ Decryption Scheme

**Figure 3:**
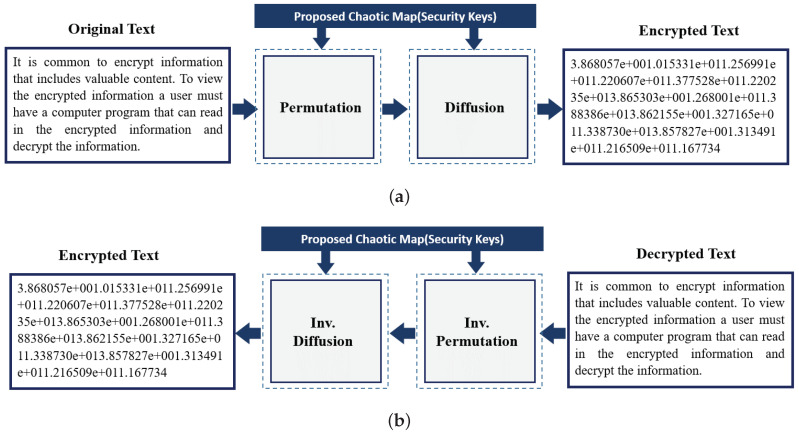
Schematic illustration of the (**a**) encryption and (**b**) decryption processes of the proposed text encryption scheme.

In recent times, text encryption is recommendable when it is transmitted or saved on insecure channels as internet. By the opposite hand, the chaotic structures have outstanding traits as mixing data, ergodicity, sensitivity to initial conditions, manipulate parameters, and so forth. In this phase, we present a symmetric text cipher algorithm primarily based on chaos. Numerous protection analysis is presented as a mystery key length, key sensitivity, frequency with histograms, auto-correlation evaluation, information entropy evaluation, differential evaluation, classic assaults evaluation, and encryption/decryption time. Structure of the our text encryption/decryption is given in Figure 3. Primarily based on numerical simulation outcomes, the proposed encryption algorithm offers excessive safety, a tremendous encryption time, and it could face up to an effective selected/regarded undeniable textual content assault; therefore, it can be implemented in real-time applications.

The design tools of the proposed textual content encryption are primarily based on more than one chaotic map with non-linear transformation capabilities. The main steps of the proposed text encryption scheme are presented in Algorithm 2.
**Algorithm 2** Key steps of the proposed text encryption scheme.**Input:** Plain-text file **$P_{t}$**.**Output:** Cipher text file **$C_{t}$**.**Begin** **Convert** the plaintext file into a one-dimensional (1-D) array, **$S_{P_{t}}$**. **Generate** the chaotic sequence by selecting two of the proposed maps (Equations (1)–(4): α=0.9, $x_{\left(0\right)}=0.1$, and $y_{\left(0\right)}=0.1$). **Change** the chaotic sequence into a uniformly distributed sequence by changing the initial values and parameters. **Permute** $S_{P_{t}}$ using the proposed chaotic map for the secret key **Create** the new vector as: $S_{P_{t}}$ = $S_{K_{t}}$ (index). **Adjust** and change $S_{P_{t}}$ utilizing the proposed chaotic map and the accompanying condition: $S_{P_{t}}\left(i\right)=\mathrm{mod}(\mathrm{round}\left(10^{12}S_{P_{t}}\left(i\right)\right),256)$. **Create** the diffused vector $S_{D_{t}}=S_{P_{t}}⊕S_{K_{t}}$, where ⊕ denotes the bit-by-bit exclusive OR operation. **Create** the final cipher text matrix $C_{t}$ = reshape ($S_{D_{t}},P_{t})$.**End**

### 2.3. The Proposed Voice Encryption /Decryption Scheme

**Figure 4:**
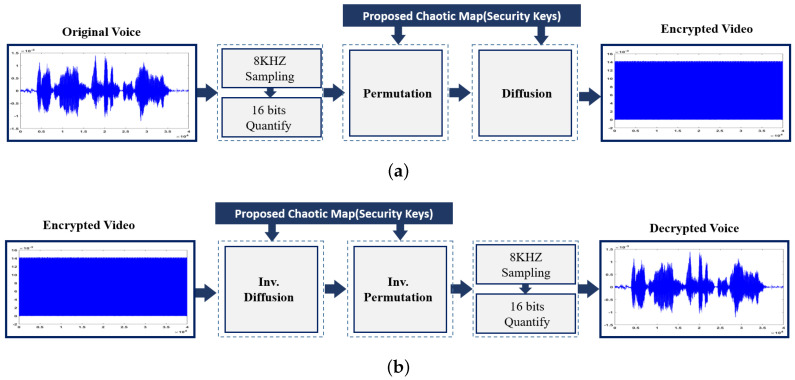
Overview of the proposed voice (**a**) encryption and (**b**) decryption steps.

The structure of the proposed scheme for voice encryption is schematized in Figure 4 and consists of two operations: permutation and masking of the speech signal using the proposed maps. The encrypted speech signal will then be sent to the receiver over a channel, which will be decrypted to recover the original speech signal according to the chaotic map.

The input to the proposed scheme is an audio file and a key. The sound file viewed as a progression of bytes. The audio header can be leaved without change so the sound data can be played again and the users still hear the scrambled sounds. Only the audio data array may be utilized as an input to the encryption strategy. The encryption set of rules scrambles and unscrambles a square length of 625 (25 × 25) bytes. The fundamental strides of the proposed encryption calculation are presented in Algorithm 3.
**Algorithm 3** Step-wise voice encryption scheme.**Input:** Plain audio file **$P_{a}$**.**Output:** Cipher audio file **$C_{a}$**.**Begin** **Load** audio data **$S_{P_{a}}$** and **Set** the control parameters ($x_{\left(0\right)}$, $y_{\left(0\right)}$, *n*, and α) to generate the key block. **Generate** the chaotic sequence. **Execute** Equations (1) and (2) to change the chaotic sequence (different chaotic maps in Figure 2 can be used). **Change** the chaotic sequence into a uniformly distributed sequence by change the initial values and parameters. **Permute** $S_{P_{a}}$ using the proposed chaotic map for the secret key **Make** the new vector as: $S_{P_{a}}$ = $S_{K_{a}}$ (index). **Adjust** and changes t$S_{P_{a}}$ utilizing the proposed chaotic map and the accompanying condition: $S_{P_{a}}\left(i\right)$= mod(round($10^{12}S_{P_{a}}\left(i\right)),256)$. **Create** the diffused vector with $S_{D_{a}}=S_{P_{a}}⊕S_{K_{a}}$, where ⊕ denotes the bit-wise XOR operation. **Create** the final cipher audio matrix $C_{a}$= reshape ($S_{D_{a}},P_{a}$).**End**

### 2.4. The Proposed Image Encryption/ Decryption Scheme

**Figure 5:**
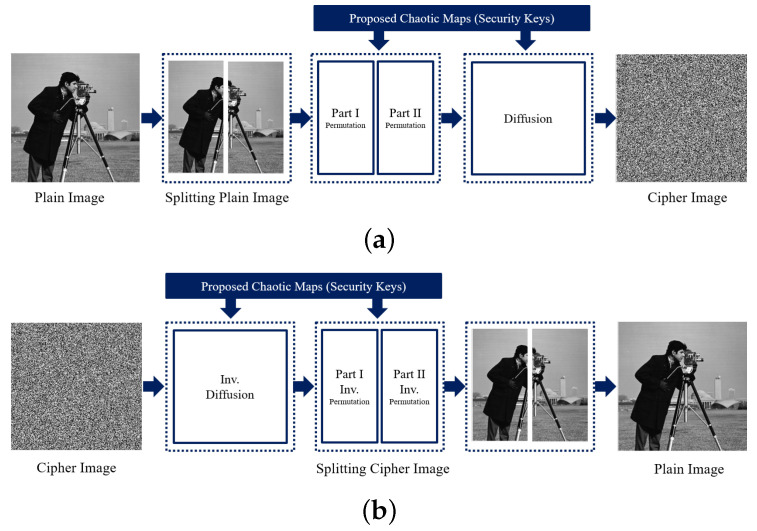
Step-wise illustration of the proposed image encryption (**a**) and decryption (**b**) scheme.

The structure of the proposed image encryption and decryption scheme is established in Figure 5. Before the predominant round, in a columns-wise direction, the pixel values in an undeniable photograph is converted into a 1D array. The latter is partitioned into two halves, the foremost and second components, each has a length of $\left(\frac{M\times N}{2}\right)$; where *M* and *N* are the image sizes. After finishing the principal spherical, an intermediate cipher photograph is gotten and is then modified into a 1D array for the next encryption round by perusing pixel values in a row-wise manner. Upon completion of the 2nd round, the final cipher photograph is obtained.

In the presented cryptosystem, two of the proposed maps are utilized. The maps are given in Equations (1)–(4) where $x_{i+1}$ and $y_{i+1}$ are state values with i=0,1,2,⋯n; and α is the parameter deciding chaotic behavior of the maps and utilized as a part of the keys within the cryptosystem. Concurring to Figure 5, the encryption details and steps are given in Algorithm 4.
**Algorithm 4** A step-by-step image encryption scheme.**Input:** Plain image **$P_{i}$**.**Output:** Cipher image **$C_{i}$**.**Begin** **Examine** the dimensions of **$P_{i}$** (i.e., *M*, *N*, grayscale or RGB color image) and **Change** it into a 1-D vector of pixels (length =M×N or 3×M×N for grayscale and color images, respectively) **Alter** the intensity values into the range (0,1) by mathematical operation included into the arrangement as the state values of the proposed mapping. **Split$P_{i}$** into two vectors ($P_{1}$ and $P_{2}$ of lengths M×N/2 each. **Generate** the chaotic sequence by selecting two of the proposed maps (e.g., **Execute** Equations (1) and (2)) **Change** the chaotic sequence (different maps in Figure 2 can be used for each half of **$P_{i}$**). **Iterate** the chaotic sequence for $P_{1}$ for scrambling $P_{1p}$ row by row and column by column (starting from the first row and the first column). **Compute** the next quantized chaotic pair using the 2nd proposed map to scramble $P_{2p}$ for $P_{2}$ with the second chaotic sequence, and reiterate this step *n* times^1^ **Combine** the two encrypted image halves (each half has its own encryption parameters) and mix the pixels of the combined image. **Permute** $S_{k_{i}}$ using the 3rd proposed map for the secret key. **Construct** the new vector of mistook pixels $S_{P_{i}}=S_{K_{i}}$ (index, size = M×N). **Adjust** and changes the vector $S_{P_{i}}$ realizing that every component of level gray ranges in [0,255] utilizing the 4-th proposed chaotic map and the accompanying condition: $S_{P_{i}}\left(i\right)=\mathrm{mod}(\mathrm{round}\left(10^{12}S_{P_{i}}\left(i\right)\right),256)$, where 1≤i≤M×N. **Create** the diffused vector $S_{D_{i}}=S_{P_{i}}⊕S_{K_{i}}$, where ⊕ denotes the bit-wise exclusive OR **Create** the final matrix with cipher image C${}_{i}$ = reshape ($S_{D_{i}}$, *M*, *N*) **Determine** the encryption image matrix and spare as **$C_{i}$**.**End**

### 2.5. The Proposed Video Encryption / Decryption Scheme

**Figure 6:**
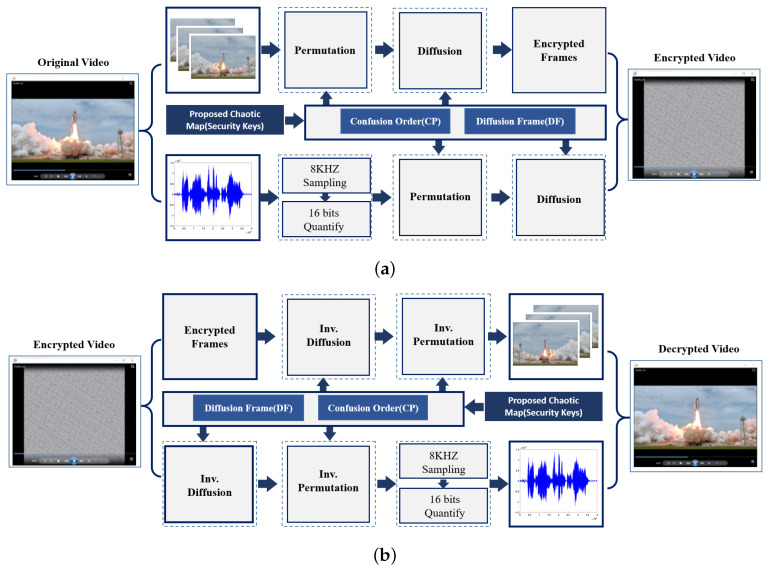
Schematic of the proposed video encryption (**a**) and decryption (**b**) stages.

The steps of the proposed technique for video encryption is schematized in Figure 6. In general, video files consists of a sequence of image frames each of which is represented as a 2D array of pixels. Video files exhibit high correlation not only for the adjacent pixels in each frame but also between successive frames. Therefore, the basis of a good cryptosystem is to devise effectual key generation procedures to decorrelate adjacent image locations. The analysis starts with separating a given video file into two parts: a sequence of image frames and voice. This image sequence is processed using the proposed image encryption (see Algorithm 4) to produce the cipher sequence. On the other hand, the voice files are processed using the proposed voice encryption method (see Algorithm 3). Then, both ciphered image and voice data are combined to produce the cipher color video frame. In total, Algorithm 5 summarizes the step-wise scheme for video encryption.
**Algorithm 5** Summary of the proposed video encryption process.**Input:** Plain Video **$P_{v}$**.**Output:** Cipher video file **$C_{v}$**.**Begin** **Separate** the image frames and voice components of the input plain video file. **Execute** Algorithm 3 for voice file encryption. **Execute** Algorithm 4 for image frames encryption. **Reverse** the operation for the decryption process to obtain **$P_{v}$**.**End**

## 3. Performance Evaluation

The quantitative performance of both traditional as well as proposed techniques could be measured through different evaluation parameters, including statistical, differential, and efficiency metrics.

### 3.1. Statistical Metrics

Good encryption should possess strong resistance against any measurable tests. The first set of these tests are statistical examinations, such as (i) histogram (ii) correlation, and (iii) information entropy analyses, should be performed [23]. Particularly, **an image histogram** describes the distribution of gray-levels of a given image. The redundancy of plaintext should be concealed in the distribution that logically needed to be uniform [23]. For a given M×N image with integer pixel intensities, q∈[0,L−1] and *L* is the number of possible grey values (usually 256), the normalized histogram ($P_{q}$) can be written by [24] as:

(5) $$P_{q}=\frac{\#\mathrm{of}\mathrm{pixels}\mathrm{with}\mathrm{intensity}q}{M\times N},q=0,1\cdots ,L-1.$$

**Cross-correlation coefficient**, termed as *R*, is another statistical metric that can quantify the randomness of encrypted images quantitatively. Mathematically, *R* between an original ($I_{o}$) and a decrypted ($I_{d}$) image can defined as [25]:

(6) $$R=\frac{\sum_{M}\sum_{N}\left(I_{o}-\bar{I_{o}}\right)\left(I_{d}-\bar{I_{d}}\right)}{\sqrt{\left(\sum_{M}\sum_{N}{\left(I_{o}-\bar{I_{o}}\right)}^{2}\right)\left(\sum_{M}\sum_{N}{\left(I_{d}-\bar{I_{d}}\right)}^{2}\right)}},$$

where $\bar{I_{o}}$ and $\bar{I_{d}}$ are the mean intensity values of original and decrypted images, respectively. Ideally, *R* value should be “1”. In addition to *R*, **information entropy** is a perfect metric for the evaluation of randomness degree. The entropy of a message source could be computed by [26] as:

(7) $$H\left(m\right)=-\sum_{i=0}^{2N_{b}-1}p\left(m_{i}\right)\log_{2}\left(p\left(m_{i}\right)\right),$$

where p(m) is the probability of symbol $m_{i}$ and $N_{b}$ is the number of bits/symbol.

### 3.2. Differential Metrics

Encrypted images needed to be sensitive to miniature modifications in the original image. The attacker can change some features in the plain image to get changes within the encrypted one. Therefore, small disturbing changes in the plain-image that induce significant changes in the encrypted version, make differential attacks useless and less effective [27]. To assess this, several metrics can be used, such as (i) mean square error (MSE) and normalized MSE [28,29]; (ii) peak signal-to-noise-ratio (PSNR) [30]; (iii) number of pixels change rate (NPCR) [30,31]; and (iv) unified average changing intensity (UACI) [32].

While the PSNR is used to measure the debasement between $I_{o}$ and $I_{d}$ [30]; NPCR is used to evaluate $I_{d}$ pixels change rate after a single pixel modification in $I_{o}$. The higher the NPCR is the more effective the performance is [29]. Practical NPCR value ought to be approximately 0.99 [32]. UACI is used to measure the average intensity of difference between plain and decrypted images [32]. Those metrics are described by Equations (8)–(11):

(8) $$MSE=\frac{1}{M\times N\times f}\sum_{k=1}^{f}\sum_{i=1}^{M}\sum_{j=1}^{N}\left(I_{o}\left(i,j,k\right)-I_{d}\left(i,j,k\right)\right)$$

(9) $$PSNR=10\log_{10}\left(\frac{I_{max}^{2}}{MSE}\right)\mathrm{dB}$$

(10) $$NPCR=100\times \frac{1}{M\times N\times f}\sum_{k=1}^{f}\sum_{i=1}^{M}\sum_{j=1}^{N}D_{i}\left(i,j,k\right)$$

$$D_{i}\left(i,j,k\right)=\left\{\begin{array}{cc} 0 & \mathrm{if}\;\;I_{o}\left(i,j,k\right)=I_{d}\left(i,j,k\right)\\ 0 & \mathrm{if}\;\;I_{o}\left(i,j,k\right)\neq I_{d}\left(i,j,k\right) \end{array}\right.$$

(11) $$UACI=100\times \frac{1}{M\times N\times f}\sum_{k=1}^{f}\sum_{i=1}^{M}\sum_{j=1}^{N}\frac{I_{o}\left(i,j,k\right)-I_{d}\left(i,j,k\right)}{2^{l}-1},$$

where *f* represents the number of frames, $I_{max}$ represents the maximum possible pixel value of the original image, and *l* is the number of bits per pixel of the original image. Please note that the normalized mean square error (NMSE) is the MSE divided by its maximum, i.e., $NMSE=\frac{MSE}{max(MSE)}$.

For audio encryption evaluation, various other metrics have been used, such as (i) signal-to-noise ratio (SNR); (ii) time-domain segemntal SNR (SNRseg); (iii) segmental spectral SNR (SSSNR) [33,34]; (iv) linear predicative code measure (LPC) [35]; and (v) cpestral distance measure (CD) [36], which are, respectively, defined mathematically as follows:

(12) $$SNR=10\log\frac{\sum_{i=1}^{L_{s}}\left|X_{\left(i\right)}\right|}{\sum_{i=1}^{L_{s}}\left(\right|X_{\left(i\right)}\left|-\right|Y_{\left(i\right)}{\left|\right)}^{2}}$$

(13) $$SNRseg=\frac{10}{N_{s}}\sum_{m=0}^{N_{s}-1}10\log_{10}\sum_{i=Nm}^{Nm+L_{s}-1}\frac{\sum_{i=1}^{N}\left|X_{\left(i\right)}\right|}{\sum_{i=1}^{L_{s}}\left(\right|X_{\left(i\right)}\left|-\right|Y_{\left(i\right)}{\left|\right)}^{2}}$$

(14) $${\left(SSSNR_{i}\right)}_{dB}=10\log\frac{\sum_{i=1}^{L_{s}}\left|X_{\left(i\right)}\right|}{\sum_{i=1}^{L_{s}}\left(\right|X_{\left(i\right)}\left|-\right|Y_{\left(i\right)}\left|\right)}$$

(15) $$d_{LPC}=\ln\left(\frac{AVA^{T}}{BVB^{T}}\right)$$

(16) $$CD=10\log_{10}\sqrt{2\sum_{n=1}^{p}{\left(C_{x}\left(n\right)-C_{y}\left(n\right)\right)}^{2}},$$

where

- $N_{s}$ is the number of segments in the output signal, and $L_{s}$ is the length of each segment; and $X_{\left(i\right)}$ and $Y_{\left(i\right)}$ are the discrete Fourier transform (DFT) of original and recovered signal, respectively, for the *i*-th speech sample.
- V is the autocorrelation matrix of the original speech block; and A and B are the LPC coefficients vectors for the clear and recovered (or encrypted) speech blocks, respectively.
- $C_{x}\left(n\right)$ and $C_{y}\left(n\right)$ the cpestral coefficients of the original and recovered (or encrypted) speech blocks, respectively.

It is worth mentioning that the SNRseg is considered the most popular time-domain metrics as it is a good estimator for speech signal quality. Mathematically, SNRseg is defined by the mean SNR value of short segments of the output signal.

### 3.3. Efficiency Metrics

High speed and Efficiency are moreover essential problems for a profitable cryptosystem, mainly in real-time applications. In general, encryption pace identity fantastically established on the Central Processing Unit (CPU)/Microprocessing Unit (MPU) structure, Random-Access Memory (RAM) size, the underlying operating system, and the programming language and its compiler options. Thus, to effectively examine the encryption complexity of two ciphers media produced on two different machines is to use the normal execution time for encryption/decryption put together, for example in milliseconds (msec) [23]. Another frequent metric to evaluate the encryption/decryption speed is bytes/sec.

## 4. Experimental Results and Discussion

Most encryption schemes are evaluated using measurable examinations to quantify a relation between both encrypted and original media. All of our simulation experiments have been conducted using MATLAB R2017a programming environment running on a Windows 7 machine, with the following specification: core i5-2400, 4 GB RAM, and a 160 GB Hard Disk Drive (HDD). All simulations/tests have been conducted more than one time. Reported times represent the average elapsed time of all trials. Comparisons with other state-of-the-art literature schemes have been conducted and evaluation of the comparative results are assessed using the above-mentioned metrics.

### 4.1. Text Simulation Results

The encrypted text is obtained with the help of the nonlinear function presented in Algorithm 2 and it is sent to the communication channel. For the decryption, the data obtained from the communication channel is transferred to the chaos generator and the inverse of the nonlinear function. Correct decryption is achievable if the chaos generator used for both encryption and decryption is the same.

**Figure 7:**
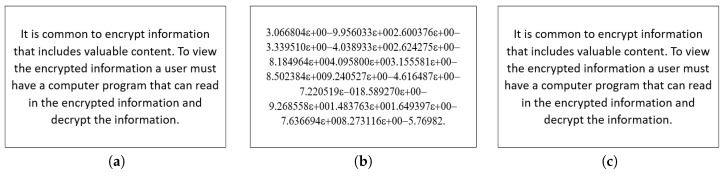
Step-by-step illustration of the text simulation results: (**a**) plain (**b**) encrypted, and (**c**) decrypted text data.

For the encryption, the plaintext in the Figure 3 is used. In Figure 7b, the encrypted text whose cipher is generated with the aid of the usage of proposed maps is shown. The decrypted text with the aid of inverting the nonlinear equation and with the assist of chaos generator is shown in Figure 7c. It is worth mentioning that the average processing time for our text encryption/decryption scheme is 20 msec (1.6 MB/sec).

**Figure 8:**
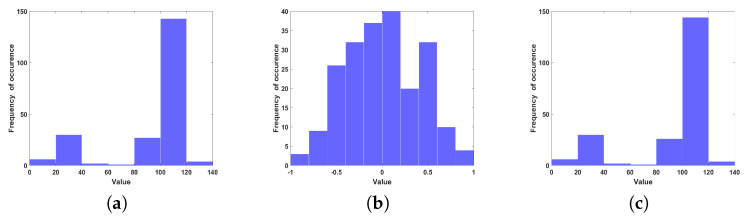
Exemplary histogram for a plain (**a**) encrypted (**b**) and decrypted (**c**) text data.

The statistical analysis of the plaintext and the encrypted version can be evaluated by histogram analysis. The histogram can offer information to find the plaintext, the secret key, or both. Just in case that the histogram content is reasonably equally circulated over the scale, no data about the plaintext can be accumulated through histogram examination. The histogram of the plaintext characters and the cipher text are shown in Figure 8a,b, respectively. As can readily seen, the cipher text histogram is uniform, so the proposed scheme is powerful against histogram attacks in addition to frequency attacks.

In addition to visual evaluation using histogram analysis, Table 1 summarizes the results of NPCR and UACI metrics for the proposed approach compared with other literature techniques. As demonstrated in the table, our scheme has achieved average values of 99.33%, and 33.42% for NPCR and UACI, respectively, which documents that the proposed algorithm is robust against differential attacks.

### 4.2. Voice Simulation Results

**Figure 9:**
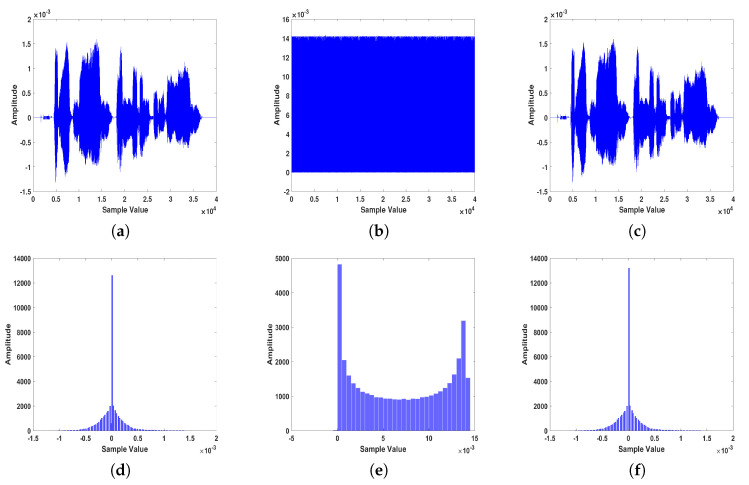
Sample example of the encryption/decryption results obtained using the proposed voice encryption approach: (**a**) original, (**b**) encrypted, and (**c**) decrypted signals. Signals’ histograms are shown in (**d**–**f**), respectively.

An example of voice simulation results using a voice block is shown in Figure 9. The voice file used in this experiment has a total length of 5 sec with a sampling frequency of 8 KHz (i.e., 40,000 samples), and an 8-bit/sample. The performance of the proposed voice system is conducted using several metrics. For example, distributions of data histogram is one of the tools used in many different fields. In encryption practices, if the distributions of numbers that represent encrypted data are close, this means encryption has been performing well. Namely, the closer the encrypted data distributions are, the higher their encryption level. The second row in Figure 9, shows the distribution versus sample value for our voice encryption approach. To statistically analyze our results, different measures that were described in Section 3), are also used for quantitative evaluation. Table 2 shows the average values of those metrics for the sample audio files analyzed using the proposed technique. In terms of processing time, the proposed scheme takes about 325 msec (2.16 MB/sec) for encryption or decryption stages.

**Figure 10:**
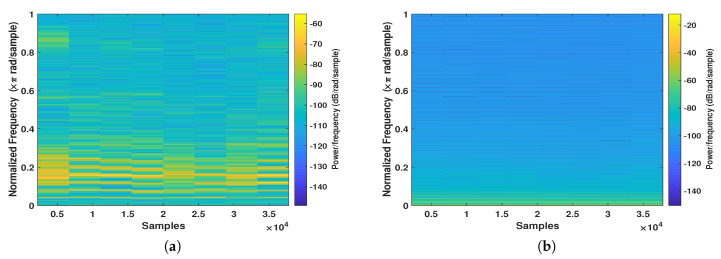
Spectrogram of the (**a**) input and (**b**) scrambled speech signals.

Moreover, Table 3 shows the quantitative comparison between the proposed scheme and state-of-the art literature voice encryption methods. As readily seen in the table, the encryption methods that use chaos outperform the traditional methods. In fact, the proposed two-level encryption scrambling scheme: scrambling and masking gives the best encryption results. The SSSNR of the main level (confused scrambling) is decreased by −7.028 dB (from 0.8823 to −6.1457) dB as contrasted with time-domain scrambling. In the subsequent level (chaotic diffusion), the SSSNR is diminished by −21.248 dB (from 0.8823 to −20.3657) dB. It additionally shows that when the two levels are consolidated, the general decrease is −26.1478 dB (from 0.8823 to −25.2655) dB. Furthermore, the key space of the proposed method is much greater than other traditional methods. The spectrograms in Figure 10 of the original and encrypted speech signals show that these two signals are quite different from higher encryption quality.

### 4.3. Image Simulation Results

**Figure 11:**
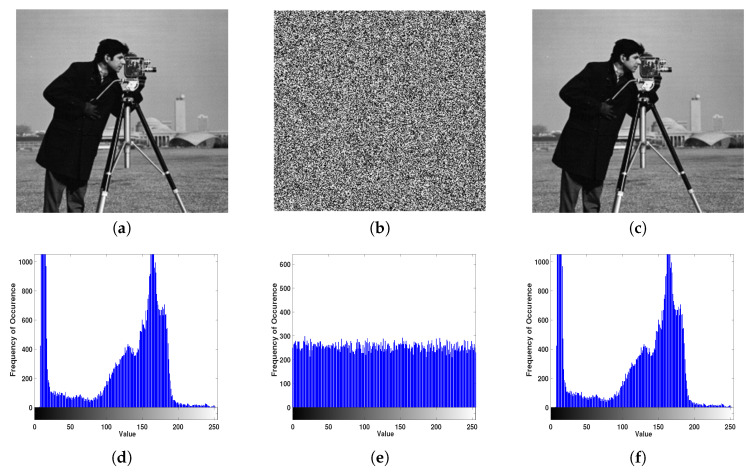
Encryption and decoding example results using the Cameraman image: (**a**) plain, (**b**) encrypted, and (**c**) decrypted images. Respective image histograms are shown in (**d**–**f**).

Sample example of the image encryption and decoding processes are depicted in Figure 11. Four reyscale images were utilized to test the proposed scheme: Lena, Cameraman, Baboon, and Pepper. The simulation results showed that the cipher images are so boisterous in a way that any data from them cannot be obtained. Table 4 summarizes the evaluation metrics for all test images. To visually evaluate our image encryption scheme, the histogram analysis is conducted. As outlined above, the nearer the distribution numbers that represent encrypted data, the better the encryption level is. The histogram for a sample image is shown in the second row of Figure 11.

The proposed encryption employments distinctive midpoints when scrambling individual input images. This, in turn, can remarkably increase the algorithm resistance to unknown or chosen attacks and differential assaults, which is a desired characteristic of cryptanalysis and secure encryption schemes. For an image encryption algorithm, NPCR bounded with UACI can measure its capacity of standing up to the differential attack. The security performance of the proposed algorithm has been conducted and compared with several state-of-the-art literature. The results can be observed in Table 5. By differentiate, the UACI (>33%) and NPCR (>99%) values obtained by our approach are very close to the perfect standard, which proves the exceedingly sensitive for our scheme for resisting differential attacks. For decryption, utilizing the proper secret keys only can correctly restore plain images from the decoded ones. The simulation results also infer that small changes made to the plain image will produce completely different images even if there’s only one bit of change between the two plain images. Additional advantage of our method is that the encryption and decryption steps take about 242 msec (1.57 MB/sec).

In addition to histogram and differential attack analyses, other well-known security analyses can be performed to support the effectiveness of a given scheme. Those analyses can be applied to any data encryption schema, however, image encryption is the most common application. Thus, we have conducted other security tests for our image encryption algorithm: key space and sensitivity, and correlation analysis.

The key space defines all-out number of various keys, which can be utilized in the encryption scheme. The proposed calculation comprises two procedures; permutation and diffusion. For permutation step, the proposed four maps are exploited with autonomous factors $x_{\left(0\right)}$, $y_{\left(0\right)}$, *a*, *b* and α for the split image and combination vector. For diffusion stage, the clench hand proposed map has independent variables $x_{\left(0\right)}$, $y_{\left(0\right)}$, *a*, *b* and α. In the key identified with the plain content algorithm, we have a consistent whole number c∈[1,255]. Thus, the key space is $\left\{x_{\left(0\right)},y_{\left(0\right)},a,b,\alpha \right\}$. Since $x_{\left(0\right)}$, $y_{\left(0\right)}$, *a*, *b* and α are two-fold accuracy numbers, their absolute number of various qualities is greater than 1014. Thus, the key space is bigger than 1014×1014×1014×1014×255 = 280,375,465,082,880 combinations of secret keys. This huge key space is sufficient to resist brute-force attacks.

**Figure 12:**
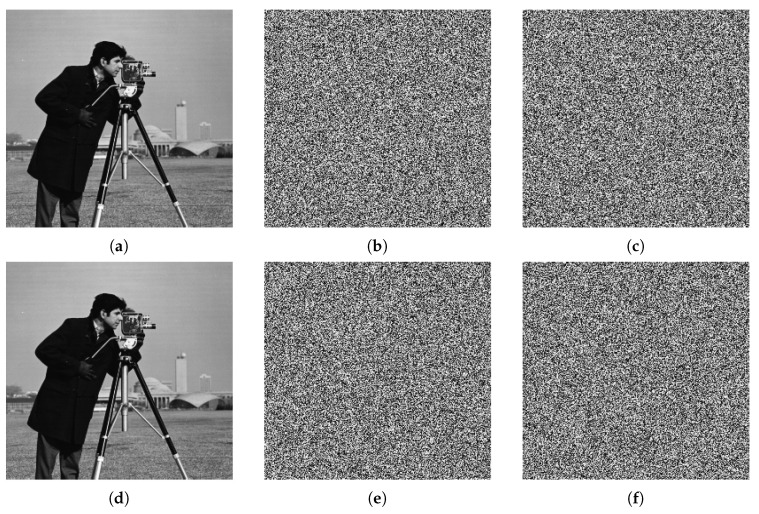
Key sensitivity results using Cameraman test image. First row is the encryption process of the plain image (**a**) and its encrypted versions with two keys (**b**,**c**). In the second row, decrypted images using correct encryption key (**d**) and wrong keys (**e**,**f**).

Key sensitivity is one of the most important features of chaos encryption. A small change in the key lead to different results during decryption, i.e., encrypted data cannot be decrypted even if only one parameter has been changed. It is also mandatory to know keys order, otherwise, the data cannot be decrypted without knowing all the keys as decryption does not happen in the correct order. Figure 12 shows encrypted images using different encrypted keys. The second row of Figure 12 demonstrates the decrypted images. Figure 12d shows the decrypted image using the same keys of correct encrypted image in Figure 12a. On the other hand, Figure 12e,f show illegal decrypted images when error/wrong keys are used. The results document that the decrypted images are all unrecognized, i.e., the original image cannot be recovered unless the correct key is used. A small change will not produce correct decryption results. Therefore, the proposed encryption algorithm has a high key sensitivity.

**Figure 13:**
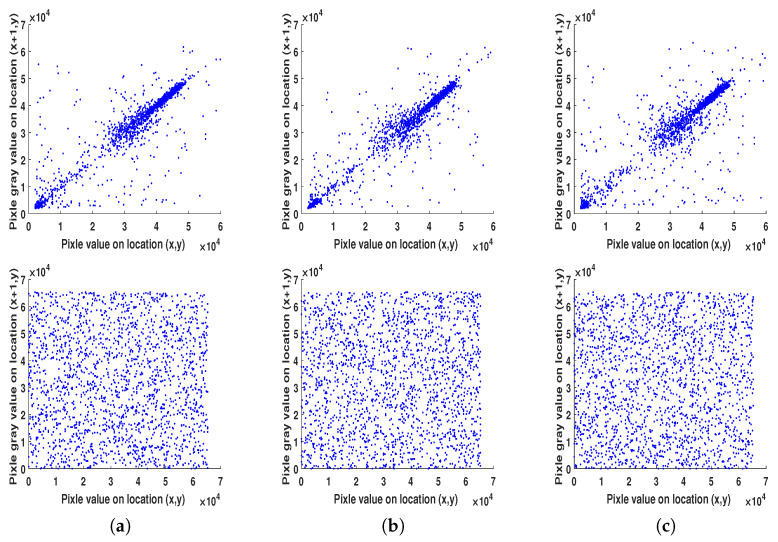
Adjacent pixel correlation analysis using Cameraman test image for the plain image (first row) and encrypted image (second row) for the horizontal (**a**), vertical (**b**), and diagonal (**c**) directions.

Another security evaluation is correlation analysis. This type of analysis visually shows the distribution between the adjacent pixels of both the original and encrypted images. Normally, plain images should exhibit strong correlations for its adjacent pixels, while the cipher images hardly have correlations for the adjacent pixels. Figure 13 demonstrates the obtained correlations in horizontal, vertical and diagonal directions for both original and encrypted images. As expected, the correlations between the adjacent pixels are very high in the original image and very low in its encrypted version in all three directions. Numerically, Table 6 summarizes the average correlation coefficients in all three directions for both plain and encrypted images. According to the results, our algorithm achieves good encryption in terms of the correlation degree of adjacent pixels (*R* values of the plain images are closer to “1” while those of the cipher images are closer to “0”), thus documenting that the proposed encryption algorithm has good confusion and diffusion properties.

### 4.4. Video Simulation Results

**Figure 14:**
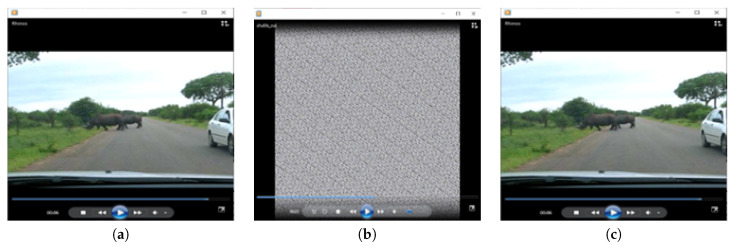
Video encryption and decoding results using Rhino.avi file: (**a**) plain, (**b**) encrypted, and (**c**) decrypted video with right keys.

The proposed video encryption scheme is used to encrypt video data, frame by frame, without considering the selective region of interest. Input color videos considered are Rhino.avi, Flamingo.avi, Train.avi, and Viptrain.avi. A one-second test video consisting of 15 frames is chosen for testing the performance of the proposed video encryption scheme and the sample video frame is shown in Figure 6. An encrypted video frame using Algorithm 5 is shown in Figure 14, which shows that the encrypted frames are unintelligible, having good perceptual security.

Differential attack refers to the method of finding out a meaningful relationship between plain and encrypted video frames by making a slight change in the pixel value of the encrypted video frame and observing its effect. If a slight change in the plain video frame results in a significant change in the cipher frame with respect to diffusion, then this encryption scheme can resist differential attack effectively. Table 7 measures the video simulation comparing the proposed approach with other literature methods.

In addition to NPCR and UACI quantitative measures, we also quantified our method based on encryption speed. In real-time the speed of encryption plays a vital role. If the time taken to encrypt/decrypt the data is high then the method may not be suitable for some applications like video conferencing, live streaming etc., The proposed method spends 1 second for creating the necessary parameters for the maps to be used and the time required to encrypt (decrypt) the test video data is about 408 msec (2.15 MB/sec). The time required to generate the keys is a one-time operation, so the proposed scheme provides a good competency as compared to its similar methods.

## 5. Conclusions

Multimedia encryption tools have become an integral part of secure and confidential data transfer over opened-nature wired or wireless communication channels. In this work, a hybrid-chaotic multimedia encryption/decryption pipeline has been proposed, which is based on novel 2D dynamical chaotic maps. The control parameters of both permutation and diffusion structure are generated by a combination of the proposed maps. Moreover, the ciphers are generated with different chaos generators for encryption and decryption and the performance analysis is conducted between these chaos generators. One of the advantages of the proposed system is its comprehensiveness in encoding all types of media (e.g., text, images, speech and video) compared with a single-secure data application. Other advantages of our system pertain to specific multimedia types. Particularly, we have presented a novel symmetric text encryption algorithm that uses plain text characteristics to resist a chosen or known plain text attack. Our proposed speech cryptosystem provided high-security features and low correlation between the original and encrypted speech signals. Moreover, the proposed image encryption/decryption scheme not only applicable for greyscale and color images but also it takes a very short time, which makes it suitable for today’s fast communication. Furthermore, the proposed video encryption possesses the ability to deal with more redundant data from the frame by frame, while providing good complexity and stability for secure encryption. In total, simulation experiments and evaluation statistics have shown that the proposed chaotic systems are faster and secure encryption/decryption of several data types with less computation as compared to conventional systems; practical in key handling; and can be implemented in real-time applications. Despite the advantages of our research, it is; however, limited in the availability of data making it difficult to process due to the limited hardware availability. Inter-operability, data processing, CPU management, memory and disk resources, and big data issues are still weaknesses in architectures that require a large number of heterogeneous devices such as Fog computing applications. There are a few research venues that are still open for future research and investigation. This includes the randomization of key choice handle, the expansion of the number of offers superimposed for increased layers’ of security; and the integration of other sorts of chaotic maps to improve the encryption handle. Another research avenue is the integration/utilization of the proposed schemes in fog applications. Generally, those applications are motivated by the desire for functionality and end-user requirements, while the security aspects are often ignored or considered as an afterthought. The impact of those security issues and possible solutions, providing future security-relevant directions will be determined for designing, developing, and maintaining fog systems.

## Figures and Tables

**Table 1:** Percentage NPCR and UACI for the text simulation comparing the proposed approach with other literature methods (“NPCR”—number of pixels change rate; and “UACI”—unified average changing intensity).

	Proposed		Ekhlas et al. [9]		Norouzi et al. [37]	
File	NPCR	UACI	NPCR	UACI	NPCR	UACI
Plain Text1	99.65	33.80	99.55	32.80	99.42	32.62
Plain Text2	98.73	33.25	98.65	33.09	99.12	33.00
Plain Text3	99.62	33.20	99.50	33.11	99.13	33.01

**Table 2:** Quantitative results of the proposed chaotic voice encryption scheme using different sample audio files. Note that “SNR”, “SNRseg”, and “R”, stand for signal-to-noise-ratio, segmental signal-to-noise-ratio, and cross correlation coefficient, respectively.

	Evaluation Metric		
Test Audio File	SNR (dB)	SNRseg (dB)	R
Sample1.wav	−41.05	−55.20	−11.80 $\times \;10^{-3}$
Sample2.wav	−48.55	−52.74	−4.00 $\times \;10^{-4}$
Sample3.wav	−43.03	−51.30	−21.00 $\times \;10^{-3}$
Sample4.wav	−45.26	−53.40	37.00 $\times \;10^{-4}$

**Table 3:** Residual intelligibility of the proposed system compared to some traditional methods. Note that “$d_{\mathrm{LPC}}$”, “SSSNR”, and “CD” stand for linear predicative code, segmental spectral signal-to-noise ratio, and cpestral distance measures, respectively.

	Evaluation Metric		
Security Scheme	$d_{\mathrm{LPC}}$	SSSNR (dB)	CD
Time Scrambling [38]	0.6532	0.9754	2.4373
Frequency Scrambling [38]	0.5723	−0.2935	2.5075
Two Dimensional Scrambling [38]	0.6732	−1.9443	3.2269
Chaotic Scrambling [38]	0.6087	−4.2272	3.4406
Chaotic Masking [38]	0.9702	−19.7036	3.8279
Two-level Encryption:(Scrambling-Masking) [38]	0.9998	−20.7803	4.2583
Proposed Chaotic system	2.0082	−25.2655	6.1201

**Table 4:** Evaluation metrics of the proposed image encryption scheme for different test images (“MSE”—mean square error; and “PSNR”—peak signal-to-noise ratio).

	Evaluation Metric		
Test Image	MSE	PSNR (dB)	Entropy
Lena	7747.31	9.24	7.9993
Cameraman	9445.44	8.38	7.9991
Baboon	7254.20	9.52	7.9993
Peppers	8413.24	8.88	7.9994
**Average**	**8215.05**	**9.01**	**7.9993**

**Table 5:** Percentage NPCR and UACI for the image simulation comparing our scheme with other literature methods (“NPCR”—number of pixels change rate; “UACI”—unified average changing intensity; and “NA”—not applicable).

	Test Image							
	Lena		Cameraman		Baboon		Peppers	
Algorithm	NPCR	UACI	NPCR	UACI	NPCR	UACI	NPCR	UACI
Wang et al. [15]	99.59	33.48	99.59	33.53	99.56	33.58	99.61	33.41
Wu et al. [16]	99.60	33.51	99.61	33.56	99.59	33.53	99.61	33.53
Amina et al. [17]	99.65	33.62	NA	NA	99.62	33.44	99.63	33.51
Luo et al. [18]	99.61	33.46	99.61	33.46	99.61	33.46	99.61	33.40
Alawida et al. [19]	99.62	33.51	NA	NA	99.60	33.42	99.62	33.39
Proposed	99.66	33.61	99.65	33.64	99.64	33.64	99.63	33.60

**Table 6:** Average correlation analysis results (%) of the proposed image encryption scheme, computed in all directions.

		Direction		
		Horizontal	Vertical	Diagonal
Test Image	Plain Image	95.09	93.47	94.01
	Encrypted Image	$33.61\times 10^{-2}$	$38.02\times 10^{-2}$	$-27.11\times 10^{-2}$

**Table 7:** Percentage NPCR, UACI for the video simulation comparing the proposed approach with other literature methods (“NPCR”—number of pixels change rate; and “UACI”—unified average changing intensity).

	Proposed		Ganeshkumar et al. [20]		Valli et al. [21]	
Test Video	NPCR	UACI	NPCR	UACI	NPCR	UACI
Rhino	99.61	33.61	99.51	33.54	99.54	33.49
Flamingo	99.63	33.63	99.52	33.52	99.58	33.48
Train	99.63	33.63	99.61	33.50	99.53	33.48
Viptrain	99.61	33.60	99.58	33.62	99.51	33.49

## Abbreviations

The following abbreviations are used in this manuscript:

1-D	One-dimensional
2-D	Two-dimensional
2D-HSM	Two-dimensional Henon-Sine map
CD	Cpestral Distance
CPU	Central Processing Unit
DDE	Delay Differential Equation
DFT	Discrete Fourier Transform
DNA	Deoxyribonucleic Acid
DRM	Digital Rights Management
HDD	Hard Disk Drive
LPC	Linear Predicative Code
MPU	Microprocessing Unit
MSE	Mean Square Error
NMSE	Normalized Mean Square Error
NPCR	Number of Pixels Change Rate
PSNR	Peak Signal-to-Noise-Ratio
PT	Processing Time
RAM	Random-Access Memory
SNR	Signal to Noise Ratio
SNRseg	Time-Domain Segemntal SNR
SSSNR	Segmental Spectral Signal to Noise Ratio
UACI	Unified Average Changing Intensity

## References

[1] Lin C.Y., Yu H.H., Zeng W. (2006). Multimedia Security Technologies for Digital Rights Management.

[2] Phillips I.E.B., Ornstein S. (2011). Securing Digital Content System and Method. U.S. Patent.

[3] Azzaz M.S., Tanougast C., Sadoudi S., Bouridane A. (2013). Synchronized hybrid chaotic generators: Application to real-time wireless speech encryption. Commun. Nonlinear Sci. Numer. Simul..

[4] Hasheminejad A., Rostami M. (2019). A novel bit level multiphase algorithm for image encryption based on PWLCM chaotic map. Optik.

[5] Yu J., Guo S., Song X., Xie Y., Wang E. (2020). Image parallel encryption technology based on sequence generator and chaotic measurement matrix. Entropy.

[6] Yousif B., Khalifa F., Makram A., Takieldeen A. (2020). A novel image encryption/decryption scheme based on integrating multiple chaotic maps. AIP Adv..

[7] Murillo-Escobar M., Meranza-Castillón M., López-Gutiérrez R., Cruz-Hernández C. (2020). A Chaotic Encryption Algorithm for Image Privacy Based on Two Pseudorandomly Enhanced Logistic Maps. Multimedia Security Using Chaotic Maps: Principles and Methodologies.

[8] Yasser I., Khalifa F., Mohamed M.A., Samrah A.S. (2020). A New Image Encryption Scheme Based on Hybrid Chaotic Maps. Complexity.

[9] Albhrany E.A., Jalil L.F., Saleh H.H. (2016). New Text Encryption Algorithm Based on Block Cipher and Chaotic Maps. Int. J. Sci. Res. Sci. Eng. Technol. (IJSRSET).

[10] Murillo-Escobar M., Abundiz-Pérez F., Cruz-Hernández C., López-Gutiérrez R. A novel symmetric text encryption algorithm based on logistic map. Proceedings of the International Conference on Communications, Signal Processing and Computers.

[11] Volos C.K., Kyprianidis I., Stouboulos I. (2013). Text Encryption Scheme Realized with a Chaotic Pseudo-Random Bit Generator. J. Eng. Sci. Technol. Rev..

[12] Yousif S.F. (2019). Speech Encryption Based on Zaslavsky Map. J. Eng. Appl. Sci..

[13] Jawad A.K., Abdullah H.N., Hreshee S.S. Secure speech communication system based on scrambling and masking by chaotic maps. Proceedings of the 2018 IEEE International Conference on Advance of Sustainable Engineering and Its Application (ICASEA).

[14] Mahdi A., Jawad A.K., Hreshee S.S. (2016). Digital chaotic scrambling of voice based on duffing map. Int. J. Inf. Commun. Sci..

[15] Wang X., Wang S., Wei N., Zhang Y. (2019). A novel chaotic image encryption scheme based on hash function and cyclic shift. IETE Tech. Rev..

[16] Wu J., Liao X., Yang B. (2018). Image encryption using 2D Hénon-Sine map and DNA approach. Signal Process..

[17] Amina S., Mohamed F.K. (2018). An efficient and secure chaotic cipher algorithm for image content preservation. Commun. Nonlinear Sci. Numer. Simul..

[18] Luo H., Ge B. (2019). Image encryption based on Henon chaotic system with nonlinear term. Multimed. Tools Appl..

[19] Alawida M., Samsudin A., Teh J.S., Alkhawaldeh R.S. (2019). A new hybrid digital chaotic system with applications in image encryption. Signal Process..

[20] Ganeshkumar D., Suresh A., Manigandan K. A New One Round Video Encryption Scheme Based on 1D Chaotic Maps. Proceedings of the 2019 IEEE 5th International Conference on Advanced Computing & Communication Systems (ICACCS).

[21] Valli D., Ganesan K. (2017). Chaos based video encryption using maps and Ikeda time delay system. Eur. Phys. J. Plus.

[22] Su S., Su Y., Xu M. (2014). Comparisons of firefly algorithm with chaotic maps. Comput. Model. New Technol..

[23] Nkapkop J.D.D., Effa J.Y., Fouda J., Alidou M., Bitjoka L., Borda M. (2014). A fast image encryption algorithm based on chaotic maps and the linear diophantine equation. Comput. Sci. Appl..

[24] Pratt W.K. (2001). Digital Image Processing.

[25] Aslantas V., Dogan A.L., Ozturk S. DWT-SVD based image watermarking using particle swarm optimizer. Proceedings of the 2008 IEEE International Conference on Multimedia and Expo.

[26] Enayatifar R. (2011). Image encryption via logistic map function and heap tree. Int. J. Phys. Sci..

[27] Gupta K., Silakari S. (2011). Efficient hybrid image cryptosystem using ECC and chaotic map. Int. J. Comput. Appl..

[28] Cox I.J., Miller M.L., Linnartz J., Kalker T. (1999). A review of watermarking principles and practices. Digit. Signal Process. Multimed. Syst..

[29] Chen E., Min L.Q., Han D.D. A chaotic system with one line equilibria and image encryption with avalanche effects. Proceedings of the 2015 International Conference on Electronics, Electrical Engineering and Information Science-EEEIS2015.

[30] Lian S., Sun J., Wang Z. (2005). A block cipher based on a suitable use of the chaotic standard map. Chaos Solitons Fractals.

[31] Feng Y., Li L., Huang F. A symmetric image encryption approach based on line maps. Proceedings of the 2006 IEEE 1st International Symposium on Systems and Control in Aerospace and Astronautics.

[32] Zhao S.Y., Guo Y.L., Dou C. Geometric imperfection effects on out-of-plane inelastic buckling loads of lateral braced arches. Proceedings of the 10th Pacific Structural Steel Conference.

[33] Abdullah H.N., Hreshee S.S., Jawad A.K. (2015). Design of Efficient noise reduction scheme for secure speech masked by chaotic signals. J. Am. Sci..

[34] Kadhim J.Q., AlAzawi M.K. (2013). Speech scrambling employing Lorenz fractional order chaotic system. J. Eng. Sustain. Dev..

[35] Strogatz S. (1994). Nonlinear Dynamics and Chaos.

[36] Sadkhab S.B., Raheema A.M., Sattar S.M.A. Design and implementation voice scrambling model based on hybrid chaotic signals. Proceedings of the International Conference of Computer and Applied Sciences (CAS).

[37] Norouzi B., Seyedzadeh S.M., Mirzakuchaki S., Mosavi M.R. (2014). A novel image encryption based on hash function with only two-round diffusion process. Multimed. Syst..

[38] Hreshee S.S., Abdullah H.N., Jawad A.K. (2018). A High Security Communication System Based on Chaotic Scrambling and Chaotic Masking. Int. J. Commun. Antenna Propag..

